# Fighting the Gender Gap in Interventional Radiology: Facts and Fiction Relating to Radiation

**DOI:** 10.1007/s00270-018-1968-2

**Published:** 2018-04-25

**Authors:** Werner Jaschke, Gabriel Bartal, Annalisa Trianni, Anna-Maria Belli

**Affiliations:** 1 Department of Radiology, Medical University of Innsbruck, Innsbruck, Austria; 2 Department of Radiology, Meir Medical Center, Street, Tchernichovsky 59, 44281 Kfar Saba, Israel; 3 Department of Physics, Udine University Hospital, Piazzale, S. Maria Della Misericordia, n. 15, 33100 Udine, Italy; 4 Radiology Department, St. George’s University Hospitals NHS Foundation Trust, London, UK

The prevalence of women radiologists has risen in the past decade [1, 2]. However, women are grossly underrepresented in Interventional Radiology [1], and this may be partly due to fear of radiation exposure, particularly in the child bearing age. There is a lot of literature on potential health effects associated with long-term exposure to low-level radiation, occupational exposure of pregnant and potentially pregnant women and radiation exposure of the conceptus [3–10]. However, practical advice for women who plan a career in interventional radiology or interventional radiologists who train and counsel female residents is rare.

The basis for the control of the occupational exposure of women who are not pregnant is the same as that for men. The additive risk of developing cancer is considered to be very small [3, 10–15]. However, women in the child bearing age may have a heightened concern about the long-term genetic risks or in case of pregnancy the risks of their unborn child related to exposure of low-level radiation. This issue has been extensively discussed in the literature [4, 9, 16, 17]. For example, according to the Report 174 of the National Council of Radiation Protection and Measurements (NCRP), there is little to no evidence among the offspring for an excess of cytogenetic syndromes, single-gene disorders, malformations, stillbirths, neonatal deaths, cancer, or cytogenetic markers that would indicate an increase in heritable genetic mutations in the exposed parents [18]. At present, there is no evidence that exposures to the conceptus below 1 mSv during the whole pregnancy involve an additional risk to the unborn child [18, 19]. Based on this threshold, guidelines were established to minimize risk to patient and conceptus from diagnostic imaging [20–22]. These guidelines help health-care workers in managing risks for pregnant patients and counseling pregnancy-related issues.

When appropriate steps are taken to establish a safe radiation environment in Interventional Radiology (IR), the occupational exposure is very low [14, 23]. Current data show that under-apron personal dose equivalent Hp(10)^1^ measurements are typically of 0.01 mSv per case for the operator; the conceptus dose is generally < 0.005 mSv per case [4]. Thus, the threshold for an increased risk to the conceptus of 1 mSv during pregnancy is not reached. Nevertheless, even if radiation dose to conceptus is lower than the limits, a strategy to reduce them to the lowest possible level has to be applied. Strategies to decrease dose to patients and staff do not differ if a potentially pregnant or pregnant IR is performing the procedure. However, the female IR should wear an apron with an equivalent of 0.5 mm Pb or higher.

## Summary

- Considering available risk evaluations, the dose limit for the conceptus of less than 1 mSv during the whole pregnancy is extremely conservative.
- Based on currently available knowledge, this limit is not reached in clinical practice and there is no need for pregnant or potentially pregnant interventional radiologists to be excluded from work on the grounds of radiation exposure.
- By keeping below the occupational dose limits, the risk of developing radiation-induced genetic defects in the offspring is negligible. Thus, women of child bearing age should not be discouraged from entering the field of IR.
- With appropriate radiation protection measures in place, the risk of developing cancer for the unborn baby and the mother is not increased.
- Robust and appropriate standardized operating procedures must be in place to prevent unintentional overexposures during fluoroscopic-guided interventions (FGI).
- Interventional radiologists should be aware of how to minimize radiation dose and when extra protective measures may be required such as in complex procedures.
- It is the woman’s choice, based on the above information and her general health during pregnancy, whether to continue to perform FGIs.

## Footnotes

1 The human body-related protection quantities, equivalent dose in an organ/tissue and effective dose, are not measurable. To overcome these practical difficulties, the International Commission on Radiation Units and Measurements (ICRU) has introduced a set of operational quantities [24–26], which can be measured and which are intended to provide a reasonable estimate for the protection quantities. The operational dose quantity used to control Effective Dose is the *Personal Dose Equivalent H*_*p*_ *(10)*. The personal dose equivalent is usually measured with a calibrated personal dosimeter worn on the body.
